# O-07 - DO WE NEED TO FOLLOW-UP SURGICALLY EXPOSED CONTACTS OF PATIENTS WITH CJD IN THE UK?

**DOI:** 10.1093/pubmed/fdag046.007

**Published:** 2026-07-09

**Authors:** Ms Katy Sinka, Ms Holly Fountain, Ms Megan Walsh

**Affiliations:** UK Health Security Agency; UK Health Security Agency; UK Health Security Agency

## Abstract

**Background:**

Surgical lookbacks for UK patients diagnosed with Creutzfeldt-Jakob disease (CJD) were standardised by the CJD Incidents Panel (2003 to 2013) and transcribed into public health guidance that still stands.. Historic surgical transmission of the prion agent underpin these public health precautions. But how real is the risk today?

**Objectives:**

Describe outcomes of the follow-up of notified patients classified as “at risk of CJD” following surgical exposure to instruments previously used on a person who later died from CJD. Consider the evidence for risk of surgically transmitted CJD in the 21st Century and whether the guidance should be changed.

**Methods:**

Patients identified through CJD surgical lookbacks were followed up to identify any associated transmission of CJD. As at the end of December 2025 person years of follow-up; years of survival post operation and causes of death for those who have died were summarised.

**Results:**

230 CJD surgical contacts, associated with 18 surgical incidents contributed 2,547 person years of follow-up time. No clinical cases of CJD were identified. Index procedures involved neurosurgery (6 brain, 2 spine), ophthalmology (2), invasive nasal surgery (5) and surgery involving lymphoid tissues in 3 patients diagnosed with variant CJD. Of the 230 surgical contacts, 83% survived at least five years post exposure (the timespan within which historic surgical transmissions became apparent). By December 2025, 84 patients had died, 17 with neurological causes of death, mainly related to the diagnosis leading to their surgical procedure. The median (range) follow-up time since operation for the remaining patients is 14.5 years (5.4 to 24 years).

**Conclusions:**

CJD surgical incidents are rare but will continue to occur. Systematic follow-up over 25 years has not identified any associated CJD transmission. The transmission risks through modern surgery are very low. The lookback and notification policy should be re-considered.

